# Weaning stress and intestinal health of piglets: A review

**DOI:** 10.3389/fimmu.2022.1042778

**Published:** 2022-11-24

**Authors:** Xiaopeng Tang, Kangning Xiong, Rejun Fang, Meijun Li

**Affiliations:** ^1^ School of Karst Science, Guizhou Normal University, State Engineering Technology Institute for Karst Desertification Control, Guiyang, China; ^2^ College of Animal Science and Technology, Hunan Agricultural University, Changsha, China; ^3^ College of Animal Science and Technology, Hunan Biological and Electromechanical Polytechnic, Changsha, China

**Keywords:** gut microbiota, inflammation, weaning stress, intestinal barrier function, intestinal development, tight junction, piglets

## Abstract

Weaning is considered to be one of the most critical periods in pig production, which is related to the economic benefits of pig farms. However, in actual production, many piglets are often subjected to weaning stress due to the sudden separation from the sow, the changes in diet and living environment, and other social challenges. Weaning stress often causes changes in the morphology and function of the small intestine of piglets, disrupts digestion and absorption capacity, destroys intestinal barrier function, and ultimately leads to reduced feed intake, increased diarrhea rate, and growth retardation. Therefore, correctly understanding the effects of weaning stress on intestinal health have important guiding significance for nutritional regulation of intestinal injury caused by weaning stress. In this review, we mainly reviewed the effects of weaning stress on the intestinal health of piglets, from the aspects of intestinal development, and intestinal barrier function, thereby providing a theoretical basis for nutritional strategies to alleviate weaning stress in mammals in future studies.

## Introduction

As the main site of nutrients digestion and absorption and an important defense line against the invasion of bacteria and endotoxins into the intestinal lumen (1, 2), the intestinal tract is an important organ in response to stress in piglets. Therefore, it is important to maintain intestinal health in animal production. However, in actual production, piglets may suffer from many stresses, such as birth stress (3), weaning stress (4), heat stress (5), transport stress (6), etc., which usually affect the intestinal health of piglets, eventually, lead to economic losses.

In modern intensive farming systems, early weaning techniques are often used to improve the productivity of sows, which can increase the annual litter size of sows, improve the utilization rate of breeding equipment, and bring more economic benefits for breeding enterprises (7). However, due to sudden separation from the sow, and the rapid changes in the diets, physical environments, and social environments, piglets may suffer from weaning stress, which jointly results in perturbation of intestinal microbiota, host physiological and biochemical functions, intestinal digestion and absorption capacity, and mucosal immune function (8, 9), which eventually leads to decrease of feed intake, occurrence of post-weaning diarrhea (PWD), and growth restriction (10, 11). That’s because, the intestinal development of the piglets is not mature, and the digestive system and immune system are not perfect at this stage, which leads to a poor ability of weaning piglets to adapt to the complex environment (11). Simultaneously, due to the insufficient secretion of digestive enzymes in the gastrointestinal tract, early-weaned piglets cannot digest solid food well, leading to the destruction of the intestinal physical barrier, including the destruction of the tight junctions (TJ), the reduction of mucins secretion, the increase of intestinal permeability and the un-balanced gut microbiota (12–14). When piglets suffered weaning stress, the intestinal environment is susceptible to invasion by pathogenic microorganisms such as *Escherichia coli* (*E. coli*), which stimulates the intestinal mucosa to secrete inflammatory factors and damage the function of the intestinal mucosal barrier (15–17). Weaning stress is not only closely related to the immune system and intestinal barrier function but also can damage the oxidation-antioxidant system and induce oxidative stress (18–20). Furthermore, weaning stress causes the dysbiosis of gut microbiota, and further increases the risk of gastrointestinal diseases in piglets (21, 22). A correct understanding of the effects of weaning stress on intestinal health has an important guiding significance for nutritional regulation of intestinal injury caused by weaning stress. Therefore, here we reviewed the effects of weaning stress on the intestinal health of piglets, from the aspects of intestinal development, and intestinal barrier function, thereby providing a theoretical basis for nutritional strategies to alleviate weaning stress in mammals in future studies.

## Weaning stress and intestinal development

### Weaning stress damages intestinal morphology

The intestines display various functions including providing a main site for nutrient digestion and absorption as well as acting as a selective barrier to prevent the entry of exogenous harmful substances into the circulation system while allowing the selective absorption of nutrients including electrolytes and water (2, 23). The integrity of the intestinal structure is the guarantee of nutrient digestion and absorption of piglets. The intestinal morphology, including villus height (VH), crypt depth (CD), and the ratio of villus height and crypt depth (VCR) reflect the health and absorption status of the intestinal function (24). The reduction in VH and VCR means that intestinal mucosal function was impaired, and intestinal digestion and absorption capacity was reduced.In contrast, a higher VH and VCR, and a lower CD of the intestines indicated better intestinal function (25, 26). As is well-known that alterations in the villus−crypt structure are universal in weaned animals, such as intestinal villus shedding, crypt hyperplasia, and intestinal mucosa atrophy, which further destroys intestinal mucosal barrier function and digestive and absorptive capacity (10, 11, 25, 27, 28). For example, a study by Bomba et al. (29) showed that, 5 days after weaning (33 days of age), the VH and VCR in the ileum of piglets were significantly lower than that before weaning (28 days of age). Similarly, Hu et al. (30) verified the deterioration of intestinal morphology induced by weaning, which showed that VH and VCR on day 3 and day 7 postweaning were decreased compared with the preweaning stage, and VH and CD did not return to preweaning levels until day 14 postweaning. Furthermore, Boudry et al. (31) reported that weaning induced long-lasting structural changes in the small intestine of piglets, which showed that the VH of the jejunum was still significantly lower on day 15 postweaning than preweaning. In addition, weaning stress has been shown to cause a decrease in the relative weight of the small intestine, with the total weight of the intestine at 15 days after weaning being only 50% of that before weaning (32). Taken together, early weaning can lead to intestinal morphological damage of piglets, including deeper CD, lower VH, reduced VCR, and lower intestinal relative weight. Therefore, to maximize pig production, it is necessary to reduce physiological changes in the small intestine caused by weaning stress.

### Weaning stress disrupts the balance between intestinal epithelial cell proliferation and apoptosis

The intestinal tract is a dynamically self-renewing tissue, and its structural and functional integrity depends on the homeostatic maintenance of the dynamic balance between proliferation and apoptosis of intestinal mucosal epithelial cells (24, 33, 34). The renewal of intestinal epithelial cells is known to be primarily involved in the proliferation of crypt stem cells, the differentiation and shedding of villus cells (35). Crypt stem cells undergo symmetric differentiation to generate stem cells to maintain self-renewal or asymmetric differentiation to generate rapidly proliferating cells (progenitor cells) (36). The progenitor cells then continuously migrate along the crypt–villus axis and finally differentiate into cells with specific functions, mainly including absorbing epithelial cells (enteroendocrine cells), Goblet cells, Endocrine cells, and Paneth cells (37, 38). Apoptosis, also known as programmed cell death, is a physiological process of cell suicide that plays an important role in the growth and development of the body (18, 33). However, due to physiological, environmental, and social challenges, piglets are easily prone to weaning stress, which disrupts the balance between cell proliferation and apoptosis of intestinal mucosal epithelial cells, and disorder of apoptosis and proliferation would increase intestinal mucosal permeability and affect intestinal barrier function (28, 39–42). Increasingly, data are available showing that the expression of genes related to proliferation and differentiation was decreased (41, 43), while the expression of genes related to apoptosis was increased in jejunal cells of weaned piglets (28, 33, 44). Montagne et al. (32) suggested that weaning disrupts the balance between intestinal cell proliferation and apoptosis in piglets, resulting in intestinal villus atrophy and crypt hyperplasia, which further leads to intestinal morphological injury. Yang et al. (45) reported that weaning-induced malnutrition in piglets would affect energy metabolism, macromolecular composition and localization, and protein metabolism, thus affecting the proliferation of intestinal crypt cells. Therefore, the reduction in intestinal cell proliferation and the increase of apoptosis caused by weaning stress may be the main mechanism responsible for intestinal mucosal injury during the postweaning period (28, 33, 41–45).

### Weaning stress inhibits the secretion of intestinal digestive enzymes

The gastrointestinal digestive enzymes are involved in the regulation of growth and development of animals, because digestive enzymes can improve the feed efficiency by digestion and in turn to modulate the process of nutrient metabolism (46). Therefore, the activities and secretion of digestive enzymes in the small intestine are important indicators to evaluate intestinal development and digestive capacity in weaned pigs (38, 47). Absorptive intestinal epithelial cells are the main cell type in the crypt-villus axis (48), which has the function of secreting a variety of digestive enzymes such as disaccharidase, peptidases, and phosphatase (49–52). In general, during the first 3 weeks after birth, the digestive system of piglets develops rapidly due to adequate nutrition intake from sows, and the activities of digestive enzymes such as intestinal lactase, protease, and lipase are significantly increased (53). However, due to the change in diet, the activities of enzymes on the brush border of the intestinal mucosa, such as disaccharidases, protease, and lipase are dramatically changed after weaning (27, 31, 38, 54, 55). It has been confirmed that intestinal morphology is associated with changes in intestinal digestive enzyme activities (14, 27, 32, 56, 57). Small intestinal disaccharidases (lactase, maltase, and sucrase) are the key enzymes of carbohydrate digestion and absorption in piglets (47). However, the activity and digestion ability of disaccharidases decreased significantly after weaning, which is considered to be an important cause of diarrhea in weaned piglets (57–59). In addition, the alkaline phosphatase (AKP) in the small intestinal villus epithelium is a landmark key enzyme associated with intestinal digestion and absorption function, which helps to increase the uptake and transport rate of nutrients, and also converts adenosine diphosphate (ADP) into adenosine triphosphate (ATP) (24, 26, 60, 61). Previous studies had demonstrated that early weaning significantly decreased small intestinal AKP activity in piglets (27, 62, 63), which indicated that weaning stress has adverse effects on intestinal digestion and absorption function. In conclusion, one possible explanation for the decrease of intestinal digestive enzymes activity in piglets caused by weaning may be due to the negative effect of weaning stress on intestinal morphology, which in turn inhibits the secretion of endogenous enzymes (51, 64, 65).

**Figure 1:**
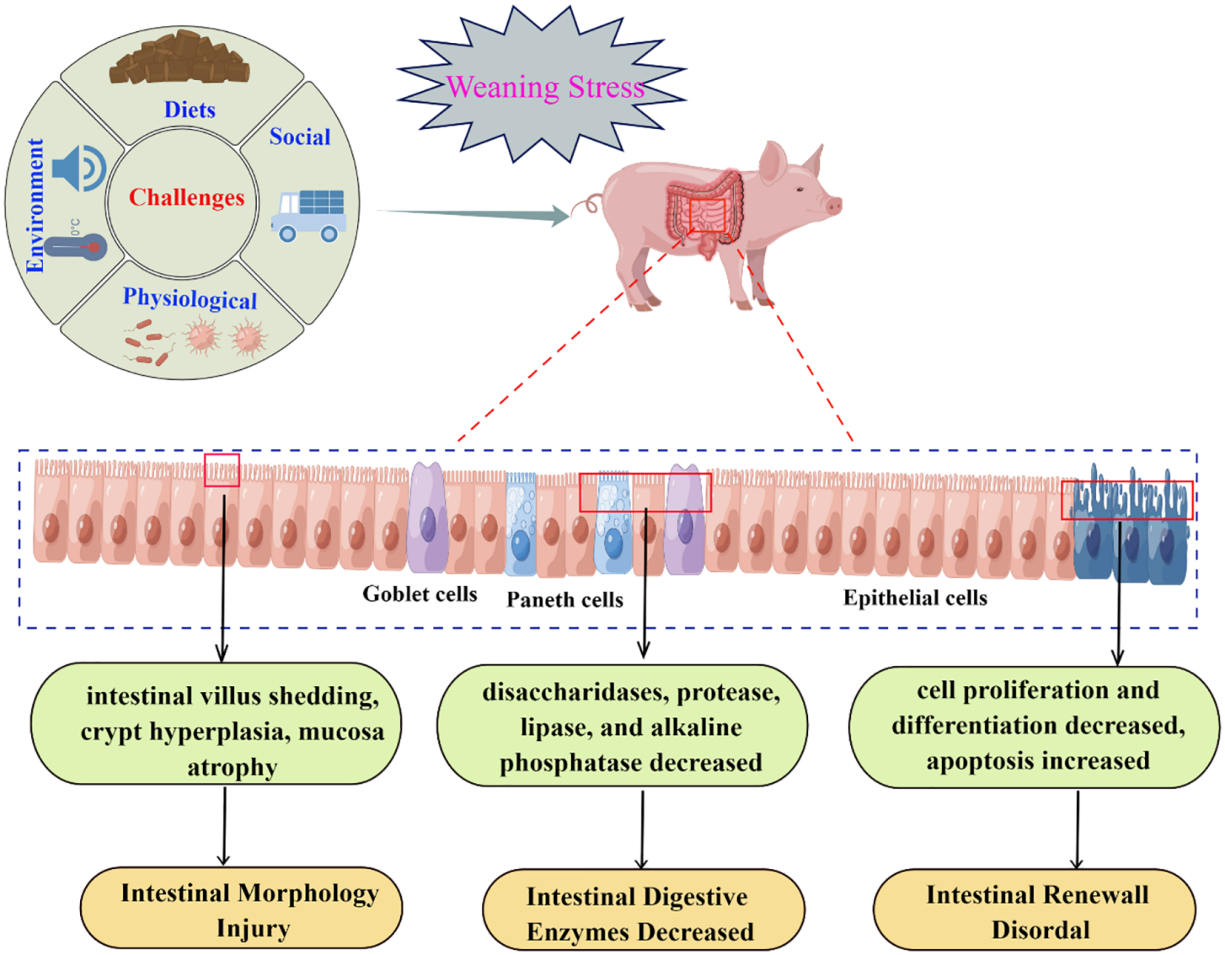
Relationship between weaning stress and intestinal development (By Figdraw).

The negative effects of weaning on the development of intestinal function can be explained as follows: first, weaning will destroy intestinal morphology and then destroy the balance between intestinal cell proliferation and apoptosis, resulting in reduced secretion of digestive enzymes; second, increased intestinal cell apoptosis further aggravated intestinal morphological damage and the decrease of digestive enzyme activity; finally, decreased digestive enzyme activity also affects intestinal morphology and apoptosis (**Figure 1**). Therefore, the decrease in digestion and absorption related enzyme activities is an important reason for the growth retardation of weaned piglets.

## Weaning stress and intestinal barrier function

The intestinal tract is constantly in contact with foreign substances, selectively absorbs effective nutrients, and resists and eliminates the invasion of toxins and enteric pathogens, and the normal operation of this mechanism depends on the integrity of intestinal barrier function (1, 24, 61). The intestinal barriers are mainly composed of intestinal epithelial cells, intestinal mucus layer, immune cells, and normal microorganisms and their metabolites, and are often artificially divided into the mechanical (physical) barrier, chemical barrier, immune barrier, and microbial barrier, which cooperate in structure and function to effectively maintain intestinal homeostasis (66–68). Separation of piglets from sows at weaning is known to induce immediate and long-term deleterious effects on gut defense mechanisms, including intestinal barrier dysfunction, intestinal inflammation, and increased intestinal permeability (30, 69–71). Intestinal barrier function was impaired at the beginning of weaning and recovered after 2 weeks of weaning. However, studies have shown that the earlier weaning occurs, the longer barrier impairment persists (71).

### Weaning stress affects the intestinal mechanical barrier

**Figure 2:**
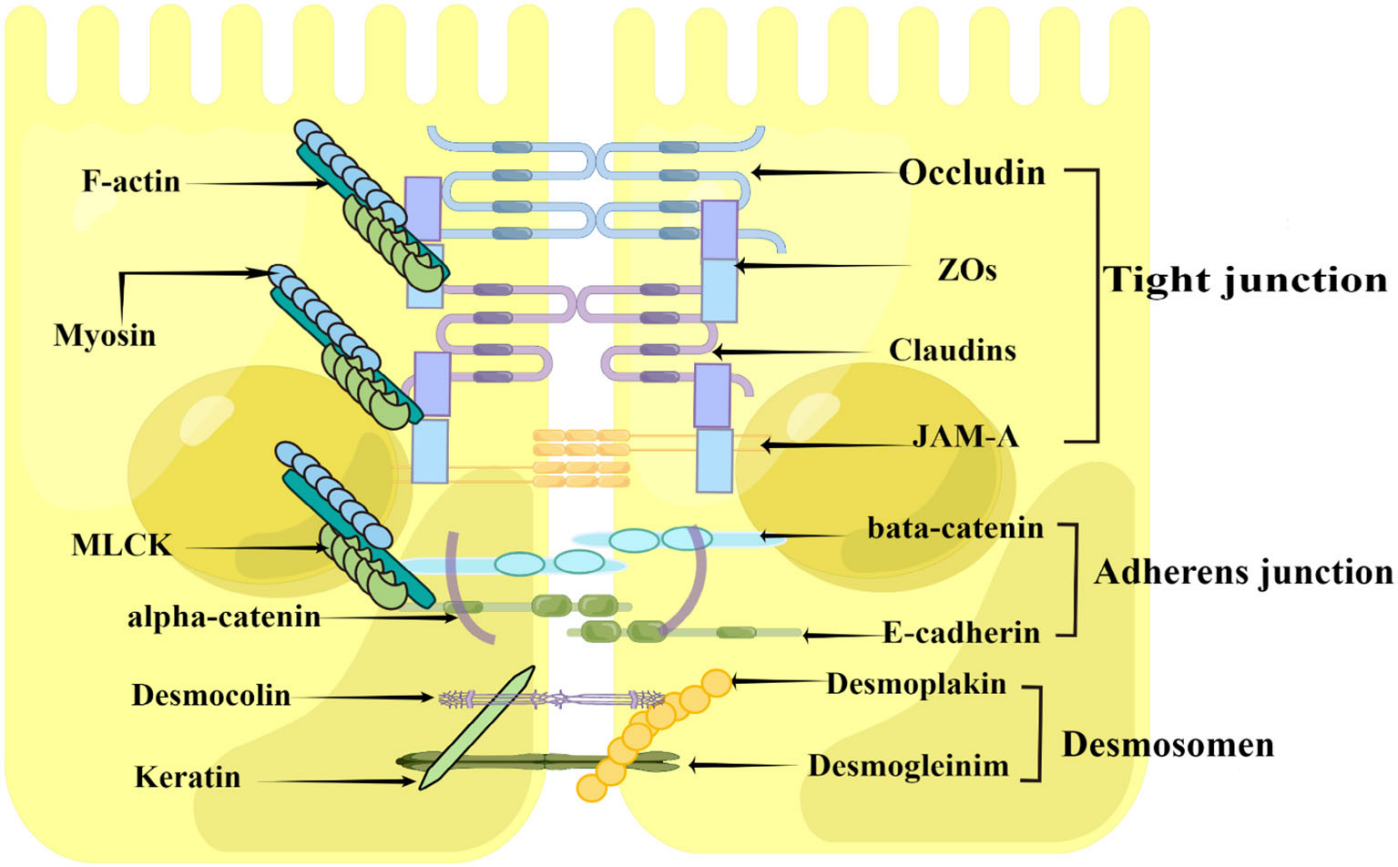
Construction of the intestinal epithelial barrier (By Figdraw). The intestinal epithelial barrier is mainly formed by a layer of epithelial cells joined together by the apical junctional complex (APC), including tight junctions, adherent junctions, and desmosomes. The tight junctions constitute the major determinant of the intestinal physical barrier, which is mainly composed of Claudin, Occludin and junctional adhesion protein molecule-A (JAM-A), zonula occludens (ZO)-1, Myosin, F-actin, and Myosin light chain kinase (MLCK).

The intestinal epithelial barrier is mainly formed by a continuous monolayer of proliferating and differentiating intestinal epithelial cells tightly linked by the apical junctional complex (AJC) (72, 73). AJC is mainly composed of TJs, adherens junctions (AJ), and desmosomes (**Figure 2**), which establishes the cellular polarity and reduces the space between adjacent cells, therefore selectively allowing the absorption of nutrients and limiting the access of pathogens, toxins, and xenobiotics from the intestinal lumen to the mucosal tissues (72, 74). Among these structures, TJs constitute the main determinant of the intestinal physical barrier (75). TJs are formed by a multiple-protein complexes located in the apical portion of the lateral membrane of intestinal epithelial cells, mainly composed of transmembrane proteins, such as Claudin, Occludin, and junctional adhesion protein molecule-A (JAM-A), Myosin, F-actin, Myosin light chain kinase (MLCK), as well as cytoplasmic proteins such as zonula occludens (ZO)-1, ZO-2 and ZO-3 (**Figure 2**), which play an important role in maintaining the intestinal mechanical barrier and regulating intestinal permeability (1, 75–77).

Previous studies have shown that weaning stress could lead to impaired physical barrier function in piglets, characterized by the disruption of intestinal epithelial TJs and increased intestinal permeability (30, 31, 71, 78). Increased intestinal permeability makes it easier for intestinal pathogenic microorganisms, endotoxins, and other antigenic substances to break the intestinal mucosal barrier and enter other tissues, organs, and the blood circulation system, which can eventually cause enteroborne infections (30, 79). In general, intestinal permeability can be reflected by the measure of transepithelial electrical resistance (TER) of the intestinal mucosa using the Ussing chamber system (4, 30, 71, 80–82). A decreased TER reflects an altered intestinal barrier (4, 82). For instance, Cao et al. (42), Hu et al. (30), Boudry et al. (31), Smith et al. (71), and Wijtten et al. (79) used Ussing chamber system to detect the intestinal TER, and the results all showed that early weaning resulted in a decreased TER in piglets, indicated that weaning stress had a destructive effect on intestinal barrier function. The detection of intestinal TJ protein expression also can be used as an important index to evaluate intestinal mechanical barrier function. Weaning stress has been established to disrupt multiple TJ proteins, including claudin-1, occluding, ZO-1 ZO-2, and ZO-3 (4, 30, 83–85), possibly by activating mitogen-activated protein kinase (MAPK) (30) and transforming growth factor-β1 (TGF-β1) signaling pathways (84), although further studies are needed to confirm this. The effects of weaning stress on the intestinal mechanical barrier function of piglets are summarized in **Table 1**. These studies suggested that weaning can damage intestinal TJ structures and increase intestinal permeability.

**Table 1:** A summary of the effects of weaning stress on intestinal mechanical barrier function in piglets.

Weaning age	Sampling time points	Significant results	References
21-d-old piglets	Piglets are killed at 0, 1, 3, and 7 d after weaning	The jejunal transepithelial electrical resistance (TER) and levels of occludin, claudin-1, and zonula occludens (ZO)-1 are decreased on d 3 and d 7 postweaning compared to d 0	Cao et al. (4)
21-d-old piglets	Piglets are killed at 25 days of age	The protein expression ofZO-1, occludin, and claudin 3 are significantly lower in the weaning piglet group (WP) group than in the suckling piglet group (SP) group	Tang et al. (28)
21-d-old piglets	Piglets are killed at 0, 3, 7, and 14 d postweaning	The jejunal TER and *occluding*, and*claudin-1* mRNA expression on d 3, 7, and 14 postweaning and *ZO-1* mRNA expression on d 3, and 7 postweaning are decreased compared to d 0	Hu et al. (30)
21-d-old piglets	Piglets are killed at 0, 2, 5, 8, or 15 d postweaning	The jejunal TER is dropped sharply, and returned to preweaning values by d 5; ileal TER increased on d 5 and is stable thereafter	Boudry et al. (31)
Piglets weaned at 15, 18, 21, 23, or 28 days of age	Piglets are slaughtered at 35 days of age	The jejunal TER is decreased in early weaning (15 to 21-day weaning age) piglets compared with that shown in late-weaned pigs (23- to 28-day weaning age)	Smith et al. (71)
21-d-old piglets	Piglets are killed at 28 days of age	The expression of occludin, claudin-1, ZO-2, and ZO-3 in the jejunum of weanling piglets are decreased compared with age-matched suckling controls	Wang et al. (83)
21-d-old piglets	Piglets are killed at 0, 3, 7, and 14 d postweaning	The level of tight junction proteins occludin and claudin-1 are reduced on d 3, 7, and 14 post-weaning, and ZO-1 protein is reduced on d 3 and d 7 postweaning	Xiao et al. (84)
21, 28, 35, and 42-d-old piglets	Piglets are killed at 56 days old	Piglets weaned at 21 days of age has a lower mRNA level of occludin and ZO-1 in jejunal and ileal mucosa, and claudin in ileal mucosa	Xun et al. (85)

### Weaning stress affects the intestinal chemical barrier

The intestinal chemical barrier is formed by the mucus layer, which is composed of mucins (MUCs) secreted by goblet cells and antimicrobial proteins secreted by epithelial cells (86, 87). The intestinal mucus layer is the first line of defense to protect intestinal epithelial cells from pathogenic microorganisms, which can effectively prevent the colonization of pathogenic microorganisms and plays an important role in maintaining the homeostasis of the intestinal environment (88–90). Mucins are glycosylated proteins with a high molecular weight characterized by an important element, the ‘mucin domain’, which comprises the main components of intestinal mucus (90, 91). Mucins can be classified into gel-forming secretory mucins (eg, MUC2, MUC5, and MUC6) and membrane-bound mucins (e.g., MUC1, MUC3, MUC4, MUC13, and MUC17) according to their structure and localization (92, 93). Among all mucins, MUC2 forms the bulk of intestinal mucus, which participates in intestinal lubrication, pathogenic bacteria antagonism, and intercellular signal transduction (61, 89, 92–95).

**Figure 3:**
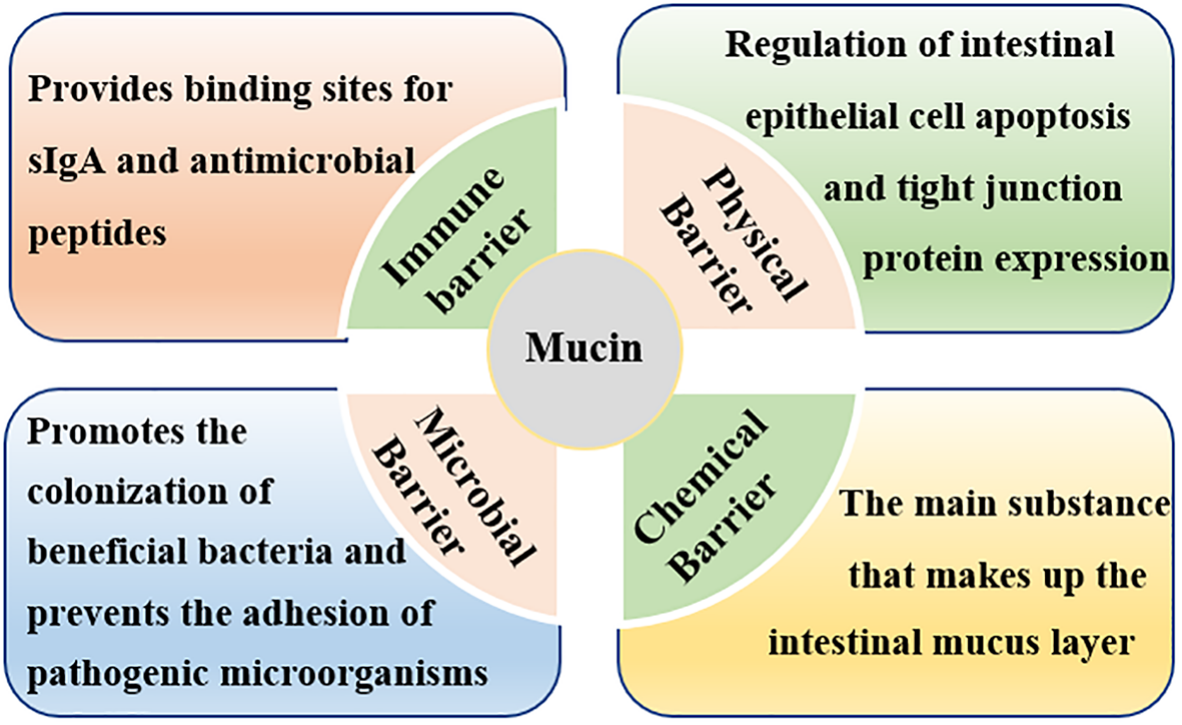
Roles of intestinal mucins in intestinal barrier function. Mucins maintain intestinal barrier function by affecting the mechanical barrier, chemical barrier, immune barrier, and microbial barrier.

Mucins are synthesized mainly by goblet cells in the gut (41, 88, 94). Therefore, any factors that affect the differentiation of goblet cells would affect the secretion of intestinal mucins. Weaning stress has been reported to injures secretory cells (mucus-producing goblet cells) differentiation and thus resulted in decreased mucins secretion (41, 96). For instance, Hedemann and Jensen (97) indicated that early weaning would not only lead to a decrease in intestinal mucin secretion but also change the glycosylation pattern of mucin, thereby weakening the intestinal chemical barrier function and increasing the probability of intestinal infection. Similarly, Yang et al. (41) reported that the *MUC2* gene was negatively regulated in weaned piglets, suggesting that weaning destructed the chemical barrier in the intestinal tract. Normal mucins secretion and expression is very important for maintaining intestinal barrier function (**Figure 3**). Firstly, when the secretion of intestinal mucins decreased, the intestinal mucosa mucus layer became thinner, and pathogenic microorganisms could easily pass through the mucus layer to destroy the function of the intestinal chemical barrier (94); secondly, invasive pathogenic microorganisms compete with the normal microbiota on the surface of the intestinal mucosa for adhesion sites, destroying the normal microbial barrier (98); thirdly, invasive pathogenic microorganisms such as *salmonella* and *shigella* could destroy the intestinal mucosal mechanical barrier by inducing apoptosis of intestinal epithelial cells as well as by disrupting the distribution of TJ proteins between intestinal mucosal cells (39, 99); finally, changed in mucins secretion and expression would cause inflammation and damage the intestinal mucosal immune barrier (100, 101).

### Weaning stress affects the intestinal immune barrier

The intestinal immune barrier is a well-developed and complex local immune system, which mainly composed of immune organs, immune cells (intraepithelial lymphocytes, lamina propria lymphocytes, neutrophils, and macrophages), and immune molecules (antibacterial peptides, immunoglobulins, and cytokines) (2, 67, 102, 103). The intestinal immune barrier is important for recognizing exogenous antigenic stimuli while ensuring that the animal body is not over-sensitive to harmless antigens (35, 104). Generally, intestinal epithelial cells recognize pathogenic molecules and beneficial substances through pattern recognition receptors (PRR), such as toll-like receptors (TLRs) and nucleotide binding oligomerization domain (NOD) like receptors (TLRs) (105, 106). It promotes the immune response and inhibits inflammation by regulating the signaling pathways of the nuclear factor kappa B (NF-κB), MAPK, and peroxisome proliferator-activated receptor γ (PPAR-γ) (107).

Conventionally, the intestinal immune system of pigs does not reach the level of adult pigs until 7 weeks of age (108, 109). However, in modern pig production, piglets are usually weaned at 3-4 weeks of age, when the intestinal immune barrier is not mature, which resulted in increased disease susceptibility (35, 109). The intestine can be regarded as the largest immune organ in animals, where up to 70% of the immune cells are localized in the mucosa and submucosa of the intestine (109, 110). Due to physiological and psychological factors, piglets would encounter many pathogenic and nonpathogenic challenges after weaning, which disrupt the immune barrier function (111, 112). Firstly, weaning resulted in intestinal immune cells, such as T lymphocytes (113, 114), and intestinal mast cell dysfunction (70, 71). McCracken et al. (113) showed that during the first 2 days postweaning, the number of intestinal inflammatory T cells and matrix metalloproteinase (i.e., stromelysin) were significantly increased; and Spreeuwenberg et al. (114) showed that the CD4+/CD8+ T lymphocytes ratio decreased after weaning, which indicated that weaning induced transient gut inflammation in pigs. Mast cell hyperactivation is an important pathophysiological mechanism in inflammatory diseases, such as irritable bowel syndrome (70, 71, 115). Studies have shown that compared with late-weaned pigs (weaned at 28 days of age), the early-weaned pigs (weaned at 19 days of age) have higher numbers of intestinal mast cells at 24 h after weaning (70) and sustained hyperplasia of intestinal mast cells at 7 weeks (116), 9 weeks (71), and 20 weeks (116) after weaning. Secondly, weaning stress activates the intestinal immune system and produces a large number of pro-inflammatory cytokines, including tumor necrosis factor-α (TNF-α), interferon-γ (IFN-γ), interleukin (IL)-1β, IL-6, IL-8 (14, 30, 117–121), and the overproduction of these cytokines can lead to intestinal damage and dysfunction (122). Thirdly, weaning leads to the destruction of intestinal integrity, microbial invasion stimulates the expression of secreted immunoglobulin A (sIgA) and defensin in the jejunum of piglets (118, 123), which is beneficial to recovering intestinal barrier function after weaning. The effects of weaning stress on the intestinal immune function of piglets are summarized in **Table 2**. These studies suggested that weaning stress can induce transient intestinal inflammation and damage the intestinal immune barrier in piglets.

**Table 2:** A summary of the effects of weaning Stress on intestinal immune function in piglets.

Weaning age	Sampling time points	Significant results	References
21-d-old piglets	Piglets are divided into Sucking group (S), Weaned group (W), and FMT + Weaned group (FW), and 4 piglets were killed at 24 days of age	mRNA expression of IL-6 and TNF-α is increased, while IL-10 is decreased in the jejunum and colon after weaning	Ma et al. (13)
28-d-old piglets	Piglets are killed at 0, 1, 2, 5, and 8 d postweaning	the levels of IL-1β, IL-6,and TNF-α are increased during the first 2 days postweaning	Pie et al. (14)
21-d-old piglets	Piglets are divided into two treatments: suckling piglet group (SP) and weaning piglet group (WP), and piglets were killed at 25 days of age	the WP group has significantly higher colonic IL-1β and lower IL-10 content than the SP group	Tang et al. (28)
21-d-old piglets	Piglets are killed at 0, 3, 7, and 14 d postweaning	mRNA levels of TNF-a and IL-6 are increased at 3 d and 7 d post-weaning	Hu et al. (30)
21-d-old piglets	Weaned and sucking piglets are killed at 25 days of age	pro-inflammatory signals (tumor necrosis factor and NO synthases 2 are increased in weaned piglets	Zhu et al. (44)
19-d-old piglets; 28-d-old piglets	Piglets are killed at twenty-four hours postweaning	the early-weaned pigs (weaned at 19 days of age) have higher numbers of intestinal mast cells than late-weaned pigs (weaned at 28 days of age)	Moeser et al. (70)
Piglets weaned at 15, 18, 21, 23, or 28 days of age	Piglets of the five groups with different weaning days are slaughtered at 35 days of age	Lamina propria immune cell density particularly mucosal mast cells is increased in early weaning (15 to 21-day weaning age) piglets	Smith et al. (71)
21-d-old piglets	Piglets are killed at 0, 0.5, 1, 2, 4, and 7 d after weaning	inflammatory T-cell numbers and local expression of matrix metalloproteinase stromelysin are increased	McCracken et al. (113)
26-d-old piglets	Piglets are killed at 0, 1, 2, and 4d after weaning	the CD4+/CD8+ T-lymphocytes ratio is decreased after weaning	Spreeuwenberg et al. (114)
16-d-old piglets; 28-d-old piglets	The pigs were killed at 7 weeks and 20 weeks	the early-weaned pigs sustained hyperplasia of intestinal mast cells at 7 weeks and 20 weeks	Pohl et al. (116)
21-d-old piglets	Piglets are killed at 0, 1, 3, 7, and 14d after weaning	TNF-α mRNA expression is enhanced from day 7 to 14; the abundance of TLR4 and IFN-γ mRNA expression are increased during the first 24 h after weaning	Deng et al. (117)
21-d-old piglets	Piglets are killed at days 0, 15, 30, and 45 postweaning	IFN-γ, IL-1α, IL-8, IL-10, IL-12α, and TGF-β in the jejunum, ileum, and colon are increased during 15 d postweaning	de Groot et al. (118)
21-d-old piglets	Piglets are sacrificed at 0, 1, 7, or 14 d after weaning	the mRNA levels of IFN-γ, iNOS, IL-6, IL-8, IL-12, and IL-22 are increased in the jejunum at 7 and 14 d after weaning	Yi et al. (119)
14-d-old piglets	Piglets are slaughtered until they were on d 0, 3, 7, 14, and 21 after weaning	early weaning disrupted IFN-γ/IL-4, IL-2/sIL-2R, and T lymphocyte balance	Cao et al. (120)
28-d-old piglets	Piglets are slaughtered at 0, 7, 14, and 21 d after weaning	the mRNA and protein expression of TNF-α and IFN-γ are increased at post-weaning day 7	Zhong et al. (121)

TNF-α, tumor necrosis factor-α; IFN-γ, interferon-γ; IL-1β, interleukin-1β; IL-2, interleukin-2; IL-4, interleukin-4; IL-6, interleukin-6; IL-8, interleukin-8; IL-10, interleukin-10; IL-12α, interleukin-12α; TLR4, toll-like receptor 4; TGF-β, transforming growth factor-β1.

### Weaning stress affects the intestinal microbial barrier

Hundreds of millions of microbiota populations inhabit the mammalian gastrointestinal tract, which play an important role in the regulation of nutrients digestion, intestinal barrier, immune response, endocrine and other physiological processes (2, 102, 124–126). It has been basically clear that dysregulation and translocation of the intestinal microbiota can cause intestinal cell apoptosis, intestinal physical barrier damage, and intestinal immune dysfunction (73, 110, 127, 128), which is not conducive to animal intestinal health. Previous studies have demonstrated that the development of the intestinal microbiota in pigs is age-dependent, with birth and weaning being the most critical periods of life (124, 129). At birth, colonization of the gut microbiota (such as *Escherichia coli* and *Streptococcus* spp.) begins once the newborn is exposed to microbes from the mother and the surrounding environment and is influenced by the consumption of colostrum and milk in sows (130). Subsequently, aerobic bacteria, facultative anaerobes, and obligate anaerobes gradually colonized the intestinal tract of piglets, and about 2 days later, the intestinal tract was completely colonized by microorganisms, and *Lactobacillus* was the dominant bacteria (17, 131).

Weaning is one of the most stressful events in piglet life, this particular period also provides an important window to shape the gut microbiota (126, 130, 132, 133). In early weaning, the diet of piglets is changed from good digestible breast milk to poor digestible solid feed, piglets cannot fully digest and utilize these nutrients due to the immature digestive system, which provides a good source of nutrients for some pathogenic bacteria to multiply, thereby altering the composition of the gut microbiota (e.g., increasing the ratio of *Escherichia coli* to *Lactobacillus*) and destroying the microbial barrier function of the intestinal tract (17, 31, 134, 135). Diarrhea is a common symptom of weaning stress, which can also lead to changes in the intestinal microbiota of piglets. Yang et al. (136) showed that the diarrheic piglets had lower relative abundances of *Bacteroides*, *Ruminococcus*, *Bulleidia*, and *Treponema*, the genera that play key roles in nutrient metabolism than healthy piglets after weaning. Similarly, Sun et al. (137) showed that the diarrheic pigs had lower relative abundances of *Bacteroidales* than non-diarrheic pigs during weaning. While the disruption of gut microbiota, in turn, aggravates post-weaning diarrhea of piglets. Of note, some pathogens can also disrupt the microbiome composition of weaned piglets (138, 139). For example, Arguello et al. (140) showed that there was an increase in pathogenic bacteria (*Citrobacter*), and a decrease in the population of beneficial bacteria such as *Bifidobacterium* and *Lactobacillus* at the ileum mucosa of weaned piglets infected with *Salmonella* Typhimurium.

**Figure 4:**
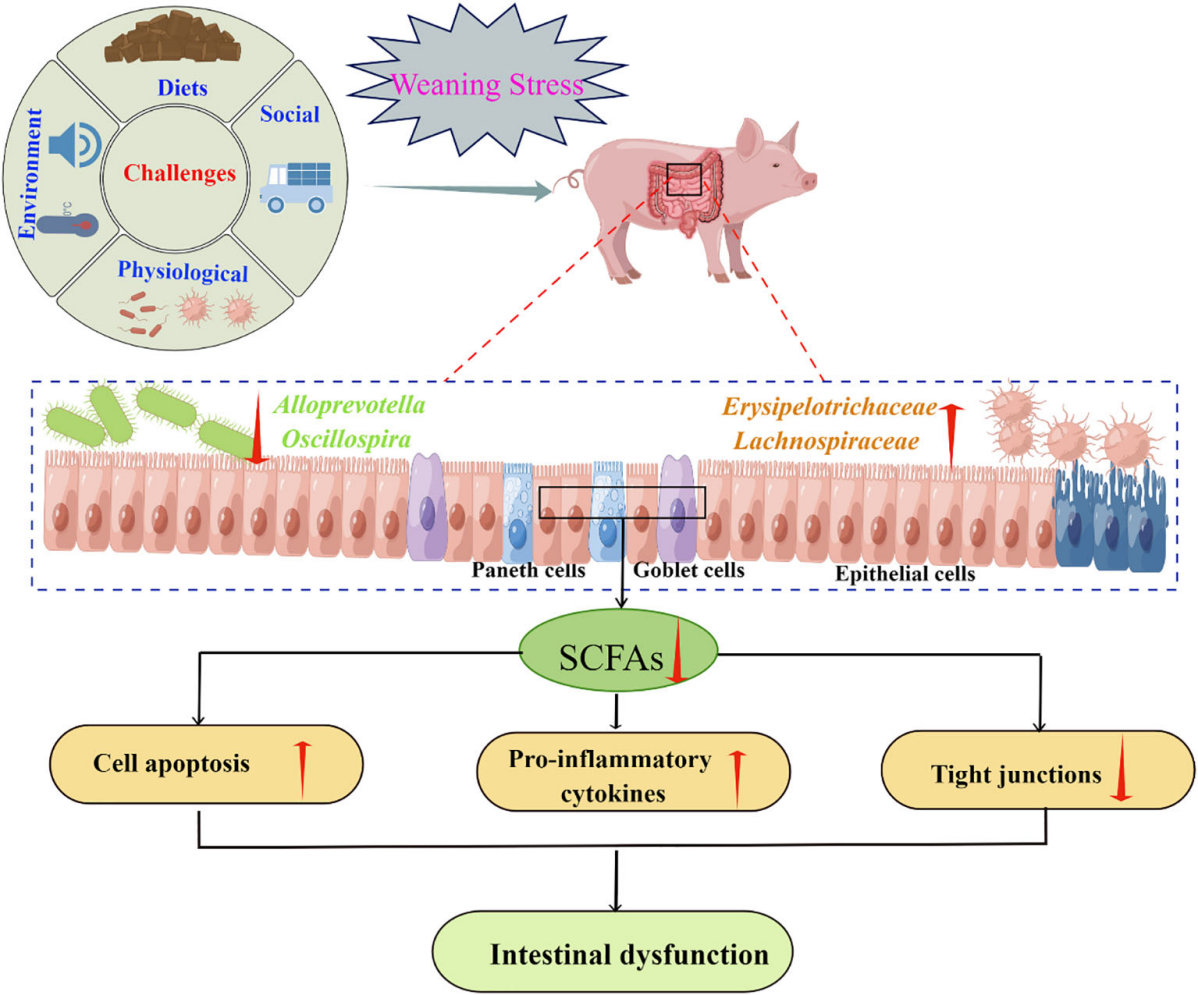
Weaning stress may affect intestinal homeostasis through microbial-driven short-chain fatty acids (SCFA) (By Figdraw). Weaning stress caused a reduction in SCFAs producing bacteria, such as *Alloprevotella* and *Oscillospira* (141), and an increase in the relative abundances of *Erysipelotrichaceae* and *Lachnospiraceaet* which is negatively correlated with butyrate production (121). SCFAs had a good regulatory effect on cell apoptosis (153), anti-inflammatory factors secretion (154), and tight junction proteins expression (155). Therefore, the intestinal dysfunction caused by weaning stress may be related to the secretion of SCFA driven by microorganisms.

Studies have shown that *Firmicutes*, *Proteobacteria*, *Bacteroidetes*, *Tenericutes*, and *Spirochaetes* were the five dominant bacterial phyla in the intestinal tract of piglets, regardless of weaning or not (141, 142). Although weaning stress generally did not change the species of the phyla, it did change the relative abundance of some phyla, especially the levels of families and genera in the corresponding phyla (143–145). For example, the abundance of *Prevotella*, a bacterium closely related to timely intake of solid feed, decreased significantly within 3 days after weaning due to weaning stress-induced feeding refusal (141, 146). However, once newly weaned piglets started to consume food, the abundance of *Prevotella* increased rapidly (144, 147, 148). Furthermore, Li et al. (141) showed that the proportion of *Alloprevotella* and *Oscillospira* decreased; and Zhong et al. (121) showed that during the first week after weaning, the relative abundances of the dominant bacterial families *Erysipelotrichaceae* and *Lachnospiraceae* increased. *Alloprevotella* has been suggested to mainly produce succinate and acetate, and the *Oscillibacter* species can produce butyrate, which plays a role in improving the intestinal barrier and exhibits anti-inflammatory function (9, 149). Butyrate production is negatively correlated with the relative abundances of *Erysipelotrichaceae* and *Lachnospiraceae* (121). Recent reports demonstrated that *Erysipelotrichaceae* has a potential role in host physiology and/or inflammation related diseases in the gastrointestinal tract (150, 151) and the increased abundance of *Lachnospiraceae* would lead to increased secretion of pro-inflammatory cytokines in the intestine (121, 152). Taken together, changes in intestinal microbiota caused by weaning stress may be related to the short-chain fatty acids (SCFA) driven by microorganisms, which might be a key modulator in the maintenance of intestinal homeostasis after weaning (**Figure 4**).

In summary, weaning stress plays a vital role in host health, which is believed to be closely associated with the function of the intestinal barrier, including the physical barrier, chemical barrier, immune barrier, and microbial barrier. First, weaning stress disrupts the intestinal mucosal mechanical barrier by reducing the expression of tight junction proteins (28, 30, 83–85), which makes it easier for bacteria and toxins to break through the intestinal mucosal barrier, and cause the release of a variety of cellular inflammatory factors, thereby leading to the occurrence of intestinal inflammation (156). Second, weaning stress disrupts the intestinal chemical barrier by inhibiting goblet cell differentiation and mucins secretion (41, 97), and decreased mucins secretion further disrupts the intestinal mechanical barrier (99), immune barrier (100), and microbial barrier (98). Third, weaning stress disrupts the intestinal chemical barrier by promoting the secretion of inflammatory factors (13, 14, 28, 30, 117–120), while pro-inflammatory cytokines including IL-1β, TNF-α, and IFN-γ, have important intestinal TJ barrier-modulating actions (157, 158). Finally, weaning stress destroys the intestinal microbial barrier by inducing intestinal microbial disorder (121, 144, 147, 148), which also adversely affects the function of the intestinal mechanical barrier (159), immune barrier (160), and chemical barrier (90).

## Conclusions

Early weaning is considered one of the most critical periods in pig production, during which the animal must face multiple stressors, such as separation from their mothers and littermates, sudden changes in feeding patterns, and environment, eventually leading to varying degrees of weaning stress symptoms. Weaning stress has a great influence on the intestinal function of piglets. At the early stage of weaning, due to the incomplete development of the intestinal function of piglets, intestinal morphology, digestive function, and the balance between apoptosis and proliferation of intestinal epithelial cells of piglets are damaged to a certain extent after sudden weaning. At the same time, weaning stress also leads to damage to the intestinal barrier function of piglets, manifested as the damage of intestinal epithelial tight junction structure; the proliferation of intestinal goblet cells and reduced secretion of intestinal mucins; increased release of intestinal pro-inflammatory factors; and increased colonization of the so called “harmful bacteria”. In conclusion, weaning stress is detrimental to gut health, but this disadvantage gradually disappears as piglets adapt to the new environment. At present, the use of nutritional interventions to alleviate stress from weaning is the mainstream research direction. Due to this, we need to correct the understanding of the effects of weaning stress on intestinal health, which is of important guiding significance for the nutritional regulation of weaning stress-induced intestinal injury.

## Author contributions

All authors listed have made a substantial, direct, and intellectual contribution to the work, and approved it for publication.

## Funding

This research was supported by grants from the China Overseas Expertise Introduction Program for Discipline Innovation (D17016); the Key Science and Technology Program of Guizhou Provence (No. 5411 2017 Qiankehe Pingtai Rencai); Guizhou Normal University Academic New Seedling Fund project (Qianshi Xinmiao [2021] B16); the Natural Science Research Project of Education Department of Guizhou Province (Qianjiaohe KY Zi [2021] 294).

## Acknowledgments

We thank all the authors who contributed references to this article, including those listed in the references and those not included in the references due to our omissions.

## Conflict of interest

The authors declare that the research was conducted in the absence of any commercial or financial relationships that could be construed as a potential conflict of interest.

## Publisher’s note

All claims expressed in this article are solely those of the authors and do not necessarily represent those of their affiliated organizations, or those of the publisher, the editors and the reviewers. Any product that may be evaluated in this article, or claim that may be made by its manufacturer, is not guaranteed or endorsed by the publisher.

## References

[1] Tang X Liu H Yang S Li Z Zhong J Fang R. Epidermal growth factor and intestinal barrier function. Mediators Inflammation (2016) 2016:1927348. doi: 10.1155/2016/1927348 PMC4976184 27524860

[2] Tang X Liu X Zhong J Fang R. Potential application of lonicera japonica extracts in animal production: From the perspective of intestinal health. Front Microbiol (2021) 12:719877. doi: 10.3389/fmicb.2021.719877 34434181 PMC8381474

[3] Gill RS Lee TF Liu JQ Chaudhary H Brocks DR Bigam DL. Cyclosporine treatment reduces oxygen free radical generation and oxidative stress in the brain of hypoxia-reoxygenated newborn piglets. PloS One (2012) 7(7):e40471. doi: 10.1371/journal.pone.0040471 22792343 PMC3392221

[4] Cao ST Wang CC Wu H Zhang QH Jiao LF Hu CH. Weaning disrupts intestinal antioxidant status, impairs intestinal barrier and mitochondrial function, and triggers mitophagy in piglets. J Anim Sci (2018) 96(3):1073–83. doi: 10.1093/jas/skx062 PMC6093500 29617867

[5] Liu F Cottrell JJ Furness JB Rivera LR Kelly FW Wijesiriwardana U. Selenium and vitamin e together improve intestinal epithelial barrier function and alleviate oxidative stress in heat-stressed pigs. Exp Physiol (2016) 101(7):801–10. doi: 10.1113/EP085746 27064134

[6] Roldan-Santiago P Trujillo-Ortega M Borderas-Tordesillas F Martínez-Rodríguez R Mora-Medina P Flores-Peinado S. Physiometabolic responses to road transport in weaned piglets for a short period and the effects of straw bedding. Anim Sci J (2015) 86(5):563–71. doi: 10.1111/asj.12324 25496132

[7] Campbell JM Crenshaw JD Polo J. The biological stress of early weaned piglets. J Anim Sci Biotechnol (2013) 4(1):19. doi: 10.1186/2049-1891-4-19 23631414 PMC3651348

[8] Yu L Li H Peng Z Ge Y Liu J Wang T. Early weaning affects liver antioxidant function in piglets. Animals (2021) 11(9):2679. doi: 10.3390/ani11092679 34573645 PMC8469846

[9] Upadhaya SD Kim IH. The impact of weaning stress on gut health and the mechanistic aspects of several feed additives contributing to improved gut health function in weanling piglets-a review. Animals (2021) 11(8):2418. doi: 10.3390/ani11082418 34438875 PMC8388735

[10] Heo JM Opapeju FO Pluske JR Kim JC Hampson DJ Nyachoti CM. Gastrointestinal health and function in weaned pigs: a review of feeding strategies to control post-weaning diarrhoea without using in-feed antimicrobial compounds. J Anim Physiol Anim Nutr (2013) 97(2):207–37. doi: 10.1111/j.1439-0396.2012.01284.x 22416941

[11] Su W Gong T Jiang Z Lu Z Wang Y. The role of probiotics in alleviating postweaning diarrhea in piglets from the perspective of intestinal barriers. Front Cell Infect Microbiol (2022) 12:883107. doi: 10.3389/fcimb.2022.883107 35711653 PMC9197122

[12] Wang J Zeng L Tan B Li G Huang B Xiong X. Developmental changes in intercellular junctions and kv channels in the intestine of piglets during the suckling and post-weaning periods. J Anim Sci Biotechnol (2016) 7:4. doi: 10.1186/s40104-016-0063-2 26819706 PMC4729073

[13] Ma X Zhang Y Xu T Qian M Yang Z Zhan X. Early-life intervention using exogenous fecal microbiota alleviates gut injury and reduce inflammation caused by weaning stress in piglets. Front Microbiol (2021) 12:671683. doi: 10.3389/fmicb.2021.671683 34177852 PMC8222923

[14] Pié S Lallès JP Blazy F Laffitte J Sève B Oswald IP. Weaning is associated with an upregulation of expression of inflammatory cytokines in the intestine of piglets. J Nutr (2004) 134(3):641–7. doi: 10.1093/jn/134.3.641 14988461

[15] Lodemann U Amasheh S Radloff J Kern M Bethe A Wieler LH. Effects of *ex vivo* infection with ETEC on jejunal barrier properties and cytokine expression in probiotic-supplemented pigs. Dig Dis Sci (2017) 62(4):922–33. doi: 10.1007/s10620-016-4413-x 27995406

[16] Ji FJ Wang LX Yang HS Hu A Yin YL. Review: The roles and functions of glutamine on intestinal health and performance of weaning pigs. Animal (2019) 13(11):2727–35. doi: 10.1017/S1751731119001800 31407650

[17] Gresse R Chaucheyras-Durand F Fleury MA Van de Wiele T Forano E Blanquet-Diot S. Gut microbiota dysbiosis in postweaning piglets: Understanding the keys to health. Trends Microbiol (2017) 25(10):851–73. doi: 10.1016/j.tim.2017.05.004 28602521

[18] Zhu LH Zhao KL Chen XL Xu JX. Impact of weaning and an antioxidant blend on intestinal barrier function and antioxidant status in pigs. J Anim Sci (2012) 90(8):2581–9. doi: 10.2527/jas.2012-4444 22896732

[19] Corino C Prost M Pizzi B Rossi R. Dietary plant extracts improve the antioxidant reserves in weaned piglets. Antioxidants (2021) 10(5):702. doi: 10.3390/antiox10050702 33946752 PMC8145250

[20] Ahasan ASML Invernizzi G Farina G Pilotto A Barbé F Bontempo V. The effects of superoxide dismutase-rich melon pulp concentrate on inflammation, antioxidant status and growth performance of challenged post-weaning piglets. Animal (2019) 13(1):136–43. doi: 10.1017/S1751731118001234 29909802

[21] Gresse R Chaucheyras-Durand F Denis S Beaumont M Van de Wiele T Forano E. Weaning-associated feed deprivation stress causes microbiota disruptions in a novel mucin-containing *in vitro* model of the piglet colon (MPigut-IVM). J Anim Sci Biotechnol (2021) 12(1):75. doi: 10.1186/s40104-021-00584-0 34078434 PMC8170946

[22] Chen L Xu Y Chen X Fang C Zhao L Chen F. The maturing development of gut microbiota in commercial piglets during the weaning transition. Front Microbiol (2017) 8:1688. doi: 10.3389/fmicb.2017.01688 28928724 PMC5591375

[23] Lallès JP Bosi P Smidt H Stokes CR. Nutritional management of gut health in pigs around weaning. Proc Nutr Soc (2007) 66(2):260–8. doi: 10.1017/S0029665107005484 17466106

[24] Tang X Xiong K. Intrauterine growth retardation affects intestinal health of suckling piglets *via* altering intestinal antioxidant capacity, glucose uptake, tight junction, and immune responses. Oxid Med Cell Longev (2022) 2022:2644205. doi: 10.1155/2022/2644205 35345830 PMC8957421

[25] Qin L Ji W Wang J Li B Hu J Wu X. Effects of dietary supplementation with yeast glycoprotein on growth performance, intestinal mucosal morphology, immune response and colonic microbiota in weaned piglets. Food Funct (2019) 10(5):2359–71. doi: 10.1039/c8fo02327a 30972390

[26] Tang X Xiong K. Epidermal growth factor activates EGFR/AMPK signalling to up-regulate the expression of SGLT1 and GLUT2 to promote intestinal glucose absorption in lipopolysaccharide challenged IPEC-J2 cells and piglets. Ital J Anim Sci (2022) 21(1):943–54. doi: 10.1080/1828051X.2022.2073832

[27] Tang M Laarveld B Van Kessel AG Hamilton DL Estrada A Patience JF. Effect of segregated early weaning on postweaning small intestinal development in pigs. J Anim Sci (1999) 77(12):3191–200. doi: 10.2527/1999.77123191x 10641863

[28] Tang W Liu J Ma Y Wei Y Liu J Wang H. Impairment of intestinal barrier function induced by early weaning *via* autophagy and apoptosis associated with gut microbiome and metabolites. Front Immunol (2021) 12:804870. doi: 10.3389/fimmu.2021.804870 34975919 PMC8714829

[29] Bomba L Minuti A Moisá SJ Trevisi E Eufemi E Lizier M. Gut response induced by weaning in piglet features marked changes in immune and inflammatory response. Funct Integr Genomics (2014) 14(4):657–71. doi: 10.1007/s10142-014-0396-x 25199657

[30] Hu CH Xiao K Luan ZS Song J. Early weaning increases intestinal permeability, alters expression of cytokine and tight junction proteins, and activates mitogen-activated protein kinases in pigs. J Anim Sci (2013) 91(3):1094–101. doi: 10.2527/jas.2012-5796 23230104

[31] Boudry G Péron V Le Huërou-Luron I Lallès JP Sève B. Weaning induces both transient and long-lasting modifications of absorptive, secretory, and barrier properties of piglet intestine. J Nutr (2004) 134(9):2256–62. doi: 10.1093/jn/134.9.2256 15333713

[32] Montagne L Boudry G Favier C Le Huërou-Luron I Lallès JP Sève B. Main intestinal markers associated with the changes in gut architecture and function in piglets after weaning. Br J Nutr (2007) 97(1):45–57. doi: 10.1017/S000711450720580X 17217559

[33] Cai X Zhu L Chen X Sheng Y Bao J Guo Q. X/XO or H2O2 induced IPEC-J2 cell as a new *in vitro* model for studying apoptosis in post-weaning piglets. Cytotechnology (2016) 68(4):713–24. doi: 10.1007/s10616-014-9823-z PMC4960122 25528136

[34] Tang X Liu B Wang X Yu Q Fang R. Epidermal growth factor, through alleviating oxidative stress, protect IPEC-J2 cells from lipopolysaccharides-induced apoptosis. Int J Mol Sci (2018) 19(3):848. doi: 10.3390/ijms19030848 29538305 PMC5877709

[35] Xiong X Yang H Hu X Wang X Li B Long L. Differential proteome analysis along jejunal crypt-villus axis in piglets. Front Biosci (Landmark Ed) (2016) 21(2):343–63. doi: 10.2741/4392 26709777

[36] Liu Y Chen YG. Intestinal epithelial plasticity and regeneration *via* cell dedifferentiation. Cell Regener (2020) 9(1):14. doi: 10.1186/s13619-020-00053-5 PMC7459029 32869114

[37] Zhou J Xiong X Wang K Zou L Lv D Yin Y. Ethanolamine metabolism in the mammalian gastrointestinal tract: Mechanisms, patterns, and importance. Curr Mol Med (2017) 17(2):92–9. doi: 10.2174/1566524017666170331161715 28429671

[38] Xiong X Tan B Song M Ji P Kim K Yin Y. Nutritional intervention for the intestinal development and health of weaned pigs. Front Vet Sci (2019) 6:46. doi: 10.3389/fvets.2019.00046 30847348 PMC6393345

[39] Souza HS Tortori CJ Castelo-Branco MT Carvalho AT Margallo VS Delgado CF. Apoptosis in the intestinal mucosa of patients with inflammatory bowel disease: evidence of altered expression of FasL and perforin cytotoxic pathways. Int J Colorectal Dis (2005) 20(3):277–86. doi: 10.1007/s00384-004-0639-8 15503066

[40] Xiao Z Liu L Tao W Pei X Wang G Wang M. Clostridium tyrobutyricum protect intestinal barrier function from LPS-induced apoptosis *via* P38/JNK signaling pathway in IPEC-J2 cells. Cell Physiol Biochem (2018) 46(5):1779–92. doi: 10.1159/000489364 29705796

[41] Yang H Xiong X Wang X Tan B Li T Yin Y. Effects of weaning on intestinal upper villus epithelial cells of piglets. PloS One (2016) 11(3):e0150216. doi: 10.1371/journal.pone 27022727 PMC4811545

[42] Yan S Long L Zong E Huang P Li J Li Y. Dietary sulfur amino acids affect jejunal cell proliferation and functions by affecting antioxidant capacity, wnt/β-catenin, and the mechanistic target of rapamycin signaling pathways in weaning piglets. J Anim Sci (2018) 96(12):5124–33. doi: 10.1093/jas/sky349 PMC6276574 30169651

[43] Wang J Chen L Li P Li X Zhou H Wang F. Gene expression is altered in piglet small intestine by weaning and dietary glutamine supplementation. J Nutr (2008) 138(6):1025–32. doi: 10.1093/jn/138.6.1025 18492829

[44] Zhu LH Xu JX Zhu SW Cai X Yang SF Chen XL. Gene expression profiling analysis reveals weaning-induced cell cycle arrest and apoptosis in the small intestine of pigs. J Anim Sci (2014) 92(3):996–1006. doi: 10.2527/jas.2013-7551 24496830

[45] Yang H Xiong X Wang X Li T Yin Y. Effects of weaning on intestinal crypt epithelial cells in piglets. Sci Rep (2016) 6:36939. doi: 10.1038/srep36939 27830738 PMC5103268

[46] Liu X Lyu W Liu L Lv K Zheng F Wang Y. Comparison of digestive enzyme activities and expression of small intestinal transporter genes in jinhua and landrace pigs. Front Physiol (2021) 12:669238. doi: 10.3389/fphys.2021.669238 34194337 PMC8236719

[47] Tang X Xiong K Wassie T Wu X. Curcumin and intestinal oxidative stress of pigs with intrauterine growth retardation: A review. Front Nutr (2022) 9:847673. doi: 10.3389/fnut.2022.847673 35571913 PMC9101057

[48] Kong S Zhang YH Zhang W. Regulation of intestinal epithelial cells properties and functions by amino acids. BioMed Res Int (2018) 2018:2819154. doi: 10.1155/2018/2819154 29854738 PMC5966675

[49] Christensen B Huber LA. The effects of creep feed composition and form and nursery diet complexity on small intestinal morphology and jejunal mucosa-specific enzyme activities after weaning in pigs. J Anim Sci (2022) 100(5):skac138. doi: 10.1093/jas/skac138 35426433 PMC9115911

[50] Fan MZ Stoll B Jiang R Burrin DG. Enterocyte digestive enzyme activity along the crypt-villus and longitudinal axes in the neonatal pig small intestine. J Anim Sci (2001) 79(2):371–81. doi: 10.2527/2001.792371x 11219446

[51] Wen JS Xu QQ Zhao WY Hu CH Zou XT Dong XY. Effects of early weaning on intestinal morphology, digestive enzyme activity, antioxidant status, and cytokine status in domestic pigeon squabs (Columba livia). Poult Sci (2022) 101(2):101613. doi: 10.1016/j.psj.2021.101613 34936957 PMC8703073

[52] Benoit YD Paré F Francoeur C Jean D Tremblay E Boudreau F. Cooperation between HNF-1alpha, Cdx2, and GATA-4 in initiating an enterocytic differentiation program in a normal human intestinal epithelial progenitor cell line. Am J Physiol Gastrointest Liver Physiol (2010) 298(4):G504–17. doi: 10.1152/ajpgi.00265.2009 PMC2907224 20133952

[53] Jensen-Waern M Melin L Lindberg R Johannisson A Petersson L Wallgren P. Dietary zinc oxide in weaned pigs–effects on performance, tissue concentrations, morphology, neutrophil functions and faecal microflora. Res Vet Sci (1998) 64(3):225–31. doi: 10.1016/s0034-5288(98)90130-8 9690608

[54] Hedemann MS Jensen BB. Variations in enzyme activity in stomach and pancreatic tissue and digesta in piglets around weaning. Arch Anim Nutr (2004) 58(1):47–59. doi: 10.1080/00039420310001656677 15085964

[55] Hedemann MS Højsgaard S Jensen BB. Small intestinal morphology and activity of intestinal peptidases in piglets around weaning. J Anim Physiol Anim Nutr (2003) 87(1-2):32–41. doi: 10.1046/j.1439-0396.2003.00405.x 14511147

[56] Wang L Yan S Li J Li Y Ding X Yin J. Rapid communication: The relationship of enterocyte proliferation with intestinal morphology and nutrient digestibility in weaning piglets. J Anim Sci (2019) 97(1):353–8. doi: 10.1093/jas/sky388 PMC6313107 30304539

[57] Marion J Petersen YM Romé V Thomas F Sangild PT Le Dividich J. Early weaning stimulates intestinal brush border enzyme activities in piglets, mainly at the posttranscriptional level. J Pediatr Gastroenterol Nutr (2005) 41(4):401–10. doi: 10.1097/01.mpg.0000177704.99786.07 16205506

[58] Kelly D King TP McFadyen M Travis AJ. Effect of lactation on the decline of brush border lactase activity in neonatal pigs. Gut (1991) 32(4):386–92. doi: 10.1136/gut.32.4.386 PMC1379076 1902807

[59] Kelly D Smyth JA McCracken KJ. Digestive development of the early-weaned pig. 1. effect of continuous nutrient supply on the development of the digestive tract and on changes in digestive enzyme activity during the first week post-weaning. Br J Nutr (1991) 65(2):169–80. doi: 10.1079/bjn19910078 1904270

[60] Tang X Liu X Zhang K. Effects of microbial fermented feed on serum biochemical profile, carcass traits, meat amino acid and fatty acid profile, and gut microbiome composition of finishing pigs. Front Vet Sci (2021) 8:744630. doi: 10.3389/fvets.2021.744630 34805337 PMC8600044

[61] Tang X Xiong K. Dietary epidermal growth factor supplementation alleviates intestinal injury in piglets with intrauterine growth retardation *via* reducing oxidative stress and enhancing intestinal glucose transport and barrier function. Animals (2022) 12:2245. doi: 10.3390/ani12172245 36077965 PMC9454730

[62] Lackeyram D Yang C Archbold T Swanson KC Fan MZ. Early weaning reduces small intestinal alkaline phosphatase expression in pigs. J Nutr (2010) 140(3):461–8. doi: 10.3945/jn.109.117267 20089775

[63] Yang C Zhu X Liu N Chen Y Gan H Troy FA. Lactoferrin up-regulates intestinal gene expression of brain-derived neurotrophic factors BDNF, UCHL1 and alkaline phosphatase activity to alleviate early weaning diarrhea in postnatal piglets. J Nutr Biochem (2014) 25(8):834–42. doi: 10.1016/j.jnutbio.2014.03.015 24824862

[64] Shang Q Ma X Liu H Liu S Piao X. Effect of fibre sources on performance, serum parameters, intestinal morphology, digestive enzyme activities and microbiota in weaned pigs. Arch Anim Nutr (2020) 74(2):121–37. doi: 10.1080/1745039X.2019.1684148 31821028

[65] Liu H Hu J Mahfuz S Piao X. Effects of hydrolysable tannins as zinc oxide substitutes on antioxidant status, immune function, intestinal morphology, and digestive enzyme activities in weaned piglets. Animals (2020) 10(5):757. doi: 10.3390/ani10050757 32349238 PMC7277717

[66] Suzuki T. Regulation of the intestinal barrier by nutrients: The role of tight junctions. Anim Sci J (2020) 91(1):e13357. doi: 10.1111/asj.13357 32219956 PMC7187240

[67] Gou HZ Zhang YL Ren LF Li ZJ Zhang L. How do intestinal probiotics restore the intestinal barrier? Front Microbiol (2022) 13:929346. doi: 10.3389/fmicb.2022.929346 35910620 PMC9330398

[68] Xu Q Jian H Zhao W Li J Zou X Dong X. Early weaning stress induces intestinal microbiota disturbance, mucosal barrier dysfunction and inflammation response activation in pigeon squabs. Front Microbiol (2022) 13:877866. doi: 10.3389/fmicb.2022.877866 35711747 PMC9194612

[69] McLamb BL Gibson AJ Overman EL Stahl C Moeser AJ. Early weaning stress in pigs impairs innate mucosal immune responses to enterotoxigenic e. coli challenge and exacerbates intestinal injury and clinical disease. PloS One (2013) 8(4):e59838. doi: 10.1371/journal.pone.0059838 23637741 PMC3634819

[70] Moeser AJ Ryan KA Nighot PK Blikslager AT. Gastrointestinal dysfunction induced by early weaning is attenuated by delayed weaning and mast cell blockade in pigs. Am J Physiol Gastrointest Liver Physiol (2007) 293(2):G413. doi: 10.1152/ajpgi.00304.2006 17525151

[71] Smith F Clark JE Overman BL Tozel CC Huang JH Rivier JE. Early weaning stress impairs development of mucosal barrier function in the porcine intestine. Am J Physiol Gastrointest Liver Physiol (2010) 298(3):G352–G363. doi: 10.1152/ajpgi.00081.2009 19926814 PMC2838512

[72] Catalioto RM Maggi CA Giuliani S. Intestinal epithelial barrier dysfunction in disease and possible therapeutical interventions. Curr Med Chem (2011) 18(3):398–426. doi: 10.2174/092986711794839179 21143118

[73] Usuda H Okamoto T Wada K. Leaky gut: Effect of dietary fiber and fats on microbiome and intestinal barrier. Int J Mol Sci (2021) 22(14):7613. doi: 10.3390/ijms22147613 34299233 PMC8305009

[74] Günzel D Yu AS. Claudins and the modulation of tight junction permeability. Physiol Rev (2013) 93(2):525–69. doi: 10.1152/physrev.00019.2012 PMC3768107 23589827

[75] Turner JR. Intestinal mucosal barrier function in health and disease. Nat Rev Immunol (2009) 9(11):799–809. doi: 10.1038/nri2653 19855405

[76] Fasano A. Physiological, pathological, and therapeutic implications of zonulin-mediated intestinal barrier modulation: living life on the edge of the wall. Am J Pathol (2008) 173(5):1243–52. doi: 10.2353/ajpath.2008.080192 PMC2570116 18832585

[77] Vancamelbeke M Vermeire S. The intestinal barrier: a fundamental role in health and disease. Expert Rev Gastroenterol Hepatol (2017) 11(9):821–34. doi: 10.1080/17474124.2017.1343143 PMC6104804 28650209

[78] Wang J Ji H. Tight junction proteins in the weaned piglet intestine: Roles and regulation. Curr Protein Pept Sci (2019) 20(7):652–60. doi: 10.2174/1389203720666190125095122 30678619

[79] Wijtten PJ Verstijnen JJ van Kempen TA Perdok HB Gort G Verstegen MW. Lactulose as a marker of intestinal barrier function in pigs after weaning. J Anim Sci (2011) 89(5):1347–57. doi: 10.2527/jas.2010-3571 21257783

[80] Tang X Fang R Pan G Xiong K. Acute effect of epidermal growth factor on phosphate diffusion across intestinal mucosa of hens using the ussing chamber system. Pakistan J Zool (2019) 51(6):2209–16. doi: 10.17582/journal.pjz/2019.51.6.2209.2216

[81] Tang X Xiong K. Effects of epidermal growth factor on glutamine and glucose absorption by IPEC-J2 cells challenged by lipopolysaccharide using the ussing chamber system. Pakistan J Zool (2021) 53(2):417–22. doi: 10.17582/journal.pjz/20200117080156

[82] Wijtten PJ van der Meulen J Verstegen MW. Intestinal barrier function and absorption in pigs after weaning: a review. Br J Nutr (2011) 105(7):967–81. doi: 10.1017/S0007114510005660 21303573

[83] Wang H Zhang C Wu G Sun Y Wang B He B. Glutamine enhances tight junction protein expression and modulates corticotropin-releasing factor signaling in the jejunum of weanling piglets. J Nutr (2015) 145(1):25–31. doi: 10.3945/jn.114.202515 25527658

[84] Xiao K Song ZH Jiao LF Ke YL Hu CH. Developmental changes of TGF-β1 and smads signaling pathway in intestinal adaption of weaned pigs. PloS One (2014) 9(8):e104589. doi: 10.1371/journal.pone.0104589 25170924 PMC4149345

[85] Xun W Shi L Zhou H Hou G Cao T. Effect of weaning age on intestinal mucosal morphology, permeability, gene expression of tight junction proteins, cytokines and secretory IgA in wuzhishan mini piglets. Ital J Anim Sci (2018) 17:976–83. doi: 10.1080/1828051X.2018.1426397

[86] Ge P Luo Y Okoye CS Chen H Liu J Zhang G. Intestinal barrier damage, systemic inflammatory response syndrome, and acute lung injury: A troublesome trio for acute pancreatitis. Biomed Pharmacother (2020) 132:110770. doi: 10.1016/j.biopha.2020.110770 33011613

[87] Johansson ME Hansson GC. Microbiology. Keeping bacteria at distance Sci (2011) 334(6053):182–3. doi: 10.1126/science.1213909 21998374

[88] Kim YS Ho SB. Intestinal goblet cells and mucins in health and disease: recent insights and progress. Curr Gastroenterol Rep (2010) 12(5):319–30. doi: 10.1007/s11894-010-0131-2 PMC2933006 20703838

[89] Pelaseyed T Bergström JH Gustafsson JK Ermund A Birchenough GM Schütte A. The mucus and mucins of the goblet cells and enterocytes provide the first defense line of the gastrointestinal tract and interact with the immune system. Immunol Rev (2014) 260(1):8–20. doi: 10.1111/imr.12182 24942678 PMC4281373

[90] Paone P Cani PD. Mucus barrier, mucins and gut microbiota: the expected slimy partners? Gut (2020) 69(12):2232–43. doi: 10.1136/gutjnl-2020-322260 PMC7677487 32917747

[91] Sicard JF Le Bihan G Vogeleer P Jacques M Harel J. Interactions of intestinal bacteria with components of the intestinal mucus. Front Cell Infect Microbiol (2017) 7:387. doi: 10.3389/fcimb.2017.00387 28929087 PMC5591952

[92] Johansson ME Hansson GC. Immunological aspects of intestinal mucus and mucins. Nat Rev Immunol (2016) 16(10):639–49. doi: 10.1038/nri.2016.88 PMC6435297 27498766

[93] Sharpe C Thornton DJ Grencis RK. A sticky end for gastrointestinal helminths; the role of the mucus barrier. Parasite Immunol (2018) 40(4):e12517. doi: 10.1111/pim.12517 29355990 PMC5900928

[94] Birchenough GM Johansson ME Gustafsson JK Bergström JH Hansson GC. New developments in goblet cell mucus secretion and function. Mucosal Immunol (2015) 8(4):712–9. doi: 10.1038/mi.2015.32 PMC4631840 25872481

[95] Ma J Rubin BK Voynow JA. Mucins, mucus, and goblet cells. Chest (2018) 154(1):169–76. doi: 10.1016/j.chest.2017.11.008 29170036

[96] Wang LX Zhu F Li JZ Li YL Ding XQ Yin J. Epidermal growth factor promotes intestinal secretory cell differentiation in weaning piglets *via* wnt/β-catenin signalling. Animal (2020) 14(4):790–8. doi: 10.1017/S1751731119002581 31650938

[97] Hedemann MS Højsgaard S Jensen BB. Lectin histochemical characterisation of the porcine small intestine around weaning. Res Vet Sci (2007) 82(2):257–62. doi: 10.1016/j.rvsc.2006.06.007 16956636

[98] Johansson ME Gustafsson JK Holmén-Larsson J Jabbar KS Xia L Xu H. Bacteria penetrate the normally impenetrable inner colon mucus layer in both murine colitis models and patients with ulcerative colitis. Gut (2014) 63(2):281–91. doi: 10.1136/gutjnl-2012-303207 PMC3740207 23426893

[99] Mirsepasi-Lauridsen HC Du Z Struve C Charbon G Karczewski J Krogfelt KA. Secretion of alpha-hemolysin by escherichia coli disrupts tight junctions in ulcerative colitis patients. Clin Transl Gastroenterol (2016) 7(3):e149. doi: 10.1038/ctg.2016.3 26938480 PMC4822097

[100] Einerhand AW Renes IB Makkink MK van der Sluis M Büller HA Dekker J. Role of mucins in inflammatory bowel disease: important lessons from experimental models. Eur J Gastroenterol Hepatol (2002) 14(7):757–65. doi: 10.1097/00042737-200207000-00008 12169985

[101] Johansson ME Sjövall H Hansson GC. The gastrointestinal mucus system in health and disease. Nat Rev Gastroenterol Hepatol (2013) 10(6):352–61. doi: 10.1038/nrgastro.2013.35 PMC3758667 23478383

[102] Qamar A Waheed J Hamza A Mohyuddin SG Lu Z Namula Z. The role of intestinal microbiota in chicken health, intestinal physiology and immunity. J Anim Plant Sci (2021) 31(2):342–51. doi: 10.36899/JAPS.2021.2.0221

[103] Wells JM Brummer RJ Derrien M MacDonald TT Troost F Cani PD. Homeostasis of the gut barrier and potential biomarkers. Am J Physiol Gastrointest Liver Physiol (2017) 312(3):G171–93. doi: 10.1152/ajpgi.00048.2015 PMC5440615 27908847

[104] Brandtzaeg P. The increasing power of immunohistochemistry and immunocytochemistry. J Immunol Methods (1998) 216(1-2):49–67. doi: 10.1016/s0022-1759(98)00070-2 9760215

[105] Bron PA van Baarlen P Kleerebezem M. Emerging molecular insights into the interaction between probiotics and the host intestinal mucosa. Nat Rev Microbiol (2011) 10(1):66–78. doi: 10.1038/nrmicro2690 22101918

[106] Wang Y Yan X Zhang W Liu Y Han D Teng K. Lactobacillus casei zhang prevents jejunal epithelial damage to early-weaned piglets induced by escherichia coli K88 *via* regulation of intestinal mucosal integrity, tight junction proteins and immune factor expression. J Microbiol Biotechnol (2019) 29(6):863–76. doi: 10.4014/jmb.1903.03054 31091863

[107] O'Flaherty S Saulnier DM Pot B Versalovic J. How can probiotics and prebiotics impact mucosal immunity? Gut Microbes (2010) 1(5):293–300. doi: 10.4161/gmic.1.5.12924m 21327037 PMC3023613

[108] Stokes CR Bailey M Haverson K Harris C Jones P Inman C. Postnatal development of intestinal immune system in piglets: implications for the process of weaning. Anim Res (2004) 53:325–34. doi: 10.1051/animres:2004020

[109] Zheng L Duarte ME Sevarolli Loftus A Kim SW. Intestinal health of pigs upon weaning: Challenges and nutritional intervention. Front Vet Sci (2021) 8:628258. doi: 10.3389/fvets.2021.628258 33644153 PMC7906973

[110] Takiishi T Fenero CIM Câmara NOS. Intestinal barrier and gut microbiota: Shaping our immune responses throughout life. Tissue Barriers (2017) 5(4):e1373208. doi: 10.1080/21688370.2017.1373208 28956703 PMC5788425

[111] Bailey M Haverson K Inman C Harris C Jones P Corfield G. The development of the mucosal immune system pre- and post-weaning: balancing regulatory and effector function. Proc Nutr Soc (2005) 64(4):451–7. doi: 10.1079/pns2005452 16313686

[112] Lauridsen C. Effects of dietary fatty acids on gut health and function of pigs pre- and post-weaning. J Anim Sci (2020) 98(4):skaa086. doi: 10.1093/jas/skaa086 32215565 PMC7323257

[113] McCracken BA Spurlock ME Roos MA Zuckermann FA Gaskins HR. Weaning anorexia may contribute to local inflammation in the piglet small intestine. J Nutr (1999) 129(3):613–9. doi: 10.1093/jn/129.3.613 10082764

[114] Spreeuwenberg MA Verdonk JM Gaskins HR Verstegen MW. Small intestine epithelial barrier function is compromised in pigs with low feed intake at weaning. J Nutr (2001) 131(5):1520–7. doi: 10.1093/jn/131.5.1520 11340110

[115] Moeser AJ Pohl CS Rajput M. Weaning stress and gastrointestinal barrier development: Implications for lifelong gut health in pigs. Anim Nutr (2017) 3(4):313–21. doi: 10.1016/j.aninu.2017.06.003 PMC5941262 29767141

[116] Pohl CS Medland JE Mackey E Edwards LL Bagley KD DeWilde MP. Early weaning stress induces chronic functional diarrhea, intestinal barrier defects, and increased mast cell activity in a porcine model of early life adversity. Neurogastroenterol Motil (2017) 29(11):e13118. doi: 10.1111/nmo.13118 PMC5650513 28573751

[117] Deng Q Tan X Wang H Wang Q Huang P Li Y. Changes in cecal morphology, cell proliferation, antioxidant enzyme, volatile fatty acids, lipopolysaccharide, and cytokines in piglets during the postweaning period. J Anim Sci (2020) 98(3):skaa046. doi: 10.1093/jas/skaa046 32047937 PMC7053866

[118] de Groot N Fariñas F Cabrera-Gómez CG Pallares FJ Ramis G. Weaning causes a prolonged but transient change in immune gene expression in the intestine of piglets. J Anim Sci (2021) 99(4):skab065. doi: 10.1093/jas/skab065 33640983 PMC8051849

[119] Yi H Jiang D Zhang L Xiong H Han F Wang Y. Developmental expression of STATs, nuclear factor-κB and inflammatory genes in the jejunum of piglets during weaning. Int Immunopharmacol (2016) 36:199–204. doi: 10.1016/j.intimp.2016.04.032 27160867

[120] Cao S Hou L Sun L Gao J Gao K Yang X. Intestinal morphology and immune profiles are altered in piglets by early-weaning. Int Immunopharmacol (2022) 105:108520. doi: 10.1016/j.intimp.2022.108520 35063748

[121] Zhong X Zhang Z Wang S Cao L Zhou L Sun A. Microbial-driven butyrate regulates jejunal homeostasis in piglets during the weaning stage. Front Microbiol (2019) 9:3335. doi: 10.3389/fmicb.2018.03335 30713531 PMC6345722

[122] Al-Sadi R Boivin M Ma T. Mechanism of cytokine modulation of epithelial tight junction barrier. Front Biosci (Landmark Ed) (2009) 14(7):2765–78. doi: 10.2741/3413 PMC3724223 19273235

[123] Ren M Huo Y Yang F Liu L Luo Y Qiao S. The changes of intestinal morphology and immune-related protein gene expressions in piglets before and after weaning. Chin J Anim Nutr (2014) 26(3):614–9. doi: 10.3969/j.issn.1006-267x.2014.03.009

[124] Zhou B Yuan Y Zhang S Guo C Li X Li G. Intestinal flora and disease mutually shape the regional immune system in the intestinal tract. Front Immunol (2020) 11:57. doi: 10.3389/fimmu.2020.0057 32318067 PMC7147503

[125] Rinninella E Raoul P Cintoni M Franceschi F Miggiano GAD Gasbarrini A. What is the healthy gut microbiota composition? a changing ecosystem across age, environment, diet, and diseases. Microorganisms (2019) 7(1):14. doi: 10.3390/microorganisms7010014 30634578 PMC6351938

[126] Beaumont M Paës C Mussard E Knudsen C Cauquil L Aymard P. Gut microbiota derived metabolites contribute to intestinal barrier maturation at the suckling-to-weaning transition. Gut Microbes (2020) 11(5):1268–86. doi: 10.1080/19490976.2020.1747335 PMC7524271 32352849

[127] Wang HX Wang YP. Gut microbiota-brain axis. Chin Med J (Engl) (2016) 129(19):2373–80. doi: 10.4103/0366-6999.190667 PMC5040025 27647198

[128] Allam-Ndoul B Castonguay-Paradis S Veilleux A. Gut microbiota and intestinal trans-epithelial permeability. Int J Mol Sci (2020) 21(17):6402. doi: 10.3390/ijms21176402 32899147 PMC7503654

[129] Karasova D Crhanova M Babak V Jerabek M Brzobohaty L Matesova Z. Development of piglet gut microbiota at the time of weaning influences development of postweaning diarrhea - a field study. Res Vet Sci (2021) 135:59–65. doi: 10.1016/j.rvsc.2020.12.022 33444908

[130] Luo Y Ren W Smidt H Wright AG Yu B Schyns G. Dynamic distribution of gut microbiota in pigs at different growth stages: Composition and contribution. Microbiol Spectr (2022) 10(3):e0068821. doi: 10.1128/spectrum.00688-21 35583332 PMC9241710

[131] Beaumont M Cauquil L Bertide A Ahn I Barilly C Gil L. Gut microbiota-derived metabolite signature in suckling and weaned piglets. J Proteome Res (2021) 20(1):982–94. doi: 10.1021/acs.jproteome.0c00745 33289566

[132] Gerzova L Babak V Sedlar K Faldynova M Videnska P Cejkova D. Characterization of antibiotic resistance gene abundance and microbiota composition in feces of organic and conventional pigs from four EU countries. PloS One (2015) 10(7):e0132892. doi: 10.1371/journal.pone.0132892 26218075 PMC4517930

[133] Kubasova T Davidova-Gerzova L Babak V Cejkova D Montagne L Le-Floc'h N. Effects of host genetics and environmental conditions on fecal microbiota composition of pigs. PloS One (2018) 13(8):e0201901. doi: 10.1371/journal.pone.0201901 30086169 PMC6080793

[134] Castillo M Martín-Orúe SM Nofrarías M Manzanilla EG Gasa J. Changes in caecal microbiota and mucosal morphology of weaned pigs. Vet Microbiol (2007) 124(3-4):239–47. doi: 10.1016/j.vetmic.2007.04.026 17532151

[135] Shin D Chang SY Bogere P Won K Choi JY Choi YJ. Beneficial roles of probiotics on the modulation of gut microbiota and immune response in pigs. PloS One (2019) 14(8):e0220843. doi: 10.1371/journal.pone.0220843 31461453 PMC6713323

[136] Yang Q Huang X Wang P Yan Z Sun W Zhao S. Longitudinal development of the gut microbiota in healthy and diarrheic piglets induced by age-related dietary changes. Microbiologyopen (2019) 8(12):e923. doi: 10.1002/mbo3.923 31496126 PMC6925166

[137] Sun J Du L Li XL Zhong H Ding Y Liu Z. Identification of the core bacteria in rectums of diarrheic and non-diarrheic piglets. Sci Rep (2019) 9:18675. doi: 10.1038/s41598-019-55328-y 31822779 PMC6904459

[138] Drumo R Pesciaroli M Ruggeri J Tarantino M Chirullo B Pistoia C. Salmonella enterica serovar typhimurium exploits inflammation to modify swine intestinal microbiota. Front Cell Infect Microbiol (2015) 5:106. doi: 10.3389/fcimb.2015.00106 26835435 PMC4722131

[139] Bescucci DM Moote PE Ortega Polo R Uwiera RRE Inglis GD. Salmonella enterica serovar typhimurium temporally modulates the enteric microbiota and host responses to overcome colonization resistance in swine. Appl Environ Microbiol (2020) 86(21):e01569–20. doi: 10.1128/AEM.01569-20 PMC7580545 32859592

[140] Argüello H Estellé J Zaldívar-López S Jiménez-Marín Á Carvajal A López-Bascón MA. Early salmonella typhimurium infection in pigs disrupts microbiome composition and functionality principally at the ileum mucosa. Sci Rep (2018) 8(1):7788. doi: 10.1038/s41598-018-26083-3 29773876 PMC5958136

[141] Li Y Guo Y Wen Z Jiang X Ma X Han X. Weaning stress perturbs gut microbiome and its metabolic profile in piglets. Sci Rep (2018) 8(1):18068. doi: 10.1038/s41598-018-33649-8 30584255 PMC6305375

[142] Hu J Nie Y Chen J Zhang Y Wang Z Fan Q. Gradual changes of gut microbiota in weaned miniature piglets. Front Microbiol (2016) 7:1727. doi: 10.3389/fmicb.2016.01727 27853453 PMC5090779

[143] Aluthge ND Van Sambeek DM Carney-Hinkle EE Li YS Fernando SC Burkey TE. The pig microbiota and the potential for harnessing the power of the microbiome to improve growth and health1. J Anim Sci (2019) 97(9):3741–57. doi: 10.1093/jas/skz208 PMC6735911 31250899

[144] Guevarra RB Lee JH Lee SH Seok MJ Kim DW Kang BN. Piglet gut microbial shifts early in life: causes and effects. J Anim Sci Biotechnol (2019) 10:1. doi: 10.1186/s40104-018-0308-3 30651985 PMC6330741

[145] Wei X Tsai T Howe S Zhao J. Weaning induced gut dysfunction and nutritional interventions in nursery pigs: A partial review. Animals (2021) 11(5):1279. doi: 10.3390/ani11051279 33946901 PMC8146462

[146] Ivarsson E Roos S Liu HY Lindberg JE. Fermentable non-starch polysaccharides increases the abundance of bacteroides-Prevotella-Porphyromonas in ileal microbial community of growing pigs. Animal (2014) 8(11):1777–87. doi: 10.1017/S1751731114001827 25046106

[147] Frese SA Parker K Calvert CC Mills DA. Diet shapes the gut microbiome of pigs during nursing and weaning. Microbiome (2015) 3:28. doi: 10.1186/s40168-015-0091-8 26167280 PMC4499176

[148] Wei X Bottoms KA Stein HH Blavi L Bradley CL Bergstrom J. Dietary organic acids modulate gut microbiota and improve growth performance of nursery pigs. Microorganisms (2021) 9(1):110. doi: 10.3390/microorganisms9010110 33466376 PMC7824888

[149] Gophna U Konikoff T Nielsen HB. Oscillospira and related bacteria - from metagenomic species to metabolic features. Environ Microbiol (2017) 19(3):835–41. doi: 10.1111/1462-2920 28028921

[150] Dinh DM Volpe GE Duffalo C Bhalchandra S Tai AK Kane AV. Intestinal microbiota, microbial translocation, and systemic inflammation in chronic HIV infection. J Infect Dis (2015) 211(1):19–27. doi: 10.1093/infdis/jiu409 25057045 PMC4326316

[151] Kaakoush NO. Insights into the role of erysipelotrichaceae in the human host. Front Cell Infect Microbiol (2015) 5:84. doi: 10.3389/fcimb.2015.00084 26636046 PMC4653637

[152] Li M Monaco MH Wang M Comstock SS Kuhlenschmidt TB Fahey GC Jr. Human milk oligosaccharides shorten rotavirus-induced diarrhea and modulate piglet mucosal immunity and colonic microbiota. ISME J (2014) 8(8):1609–20. doi: 10.1038/ismej.2014.10 PMC4817601 24522264

[153] Gui H Shen Z. Concentrate diet modulation of ruminal genes involved in cell proliferation and apoptosis is related to combined effects of short-chain fatty acid and pH in rumen of goats. J Dairy Sci (2016) 99(8):6627–38. doi: 10.3168/jds.2015-10446 27236768

[154] Bach Knudsen KE Lærke HN Hedemann MS Nielsen TS Ingerslev AK Gundelund Nielsen DS. Impact of diet-modulated butyrate production on intestinal barrier function and inflammation. Nutrients (2018) 10(10):1499. doi: 10.3390/nu10101499 30322146 PMC6213552

[155] Yan H Ajuwon KM. Butyrate modifies intestinal barrier function in IPEC-J2 cells through a selective upregulation of tight junction proteins and activation of the akt signaling pathway. PloS One (2017) 12(6):e0179586. doi: 10.1371/journal.pone.0179586 28654658 PMC5487041

[156] Li N Gu L Qu L Gong J Li Q Zhu W. Berberine attenuates pro-inflammatory cytokine-induced tight junction disruption in an *in vitro* model of intestinal epithelial cells. Eur J Pharm Sci (2010) 40(1):1–8. doi: 10.1016/j.ejps 20149867

[157] Kaminsky LW Al-Sadi R Ma TY. IL-1β and the intestinal epithelial tight junction barrier. Front Immunol (2021) 12:767456. doi: 10.3389/fimmu.2021.767456 34759934 PMC8574155

[158] Al-Sadi R Guo S Ye D Rawat M Ma TY. TNF-α modulation of intestinal tight junction permeability is mediated by NIK/IKK-α axis activation of the canonical NF-κb pathway. Am J Pathol (2016) 186(5):1151–65. doi: 10.1016/j.ajpath.2015.12.016 PMC4861759 26948423

[159] Li XY He C Zhu Y Lu NH. Role of gut microbiota on intestinal barrier function in acute pancreatitis. World J Gastroenterol (2020) 26(18):2187–93. doi: 10.3748/wjg.v26.i18.2187 PMC7235204 32476785

[160] Goto Y Panea C Nakato G Cebula A Lee C Diez MG. Segmented filamentous bacteria antigens presented by intestinal dendritic cells drive mucosal Th17 cell differentiation. Immunity (2014) 40(4):594–607. doi: 10.1016/j.immuni.2014.03.005 24684957 PMC4084624

